# How to adapt message to adolescents about sexuality?

**DOI:** 10.1192/j.eurpsy.2023.1511

**Published:** 2023-07-19

**Authors:** H. Ghabi, A. Aissa, F. Askri, S. Jedda, Y. Zgueb, U. Ouali

**Affiliations:** 1 Avicenna, Razi psychiatric hospital, Manouba, Tunisia

## Abstract

**Introduction:**

A mature and fulfilling sexuality is based on appropriate sexual education. The message must be adapted to the level of knowledge and practices of young people. Old studies dating back more than 15 years have been published.

**Objectives:**

The objective of this study is to assess adolescents’ knowledge and attitudes about sexuality.

**Methods:**

This is a descriptive cross-sectional study conducted among 80 adolescents using an anonymous online questionnaire.

**Results:**

The average age of the participants was 18 years old 45% had had at least one sexual intercourse, they are mostly male. Only 9% had used a method of contraception. Most of them had heard of contraceptive techniques. Young age, male gender, lack of dialogue with parents, low socio-economic status and lack of sex education were significantly associated with a low level of knowledge about sexuality.

**Conclusions:**

The results show that adolescents had risky practices with a lack of information. More studies are needed to approve these results and improve sexual health of these teenagers thanks to targeted sensitization.

**Disclosure of Interest:**

None Declared

